# Noxious stimulation induces self-protective behavior in bumblebees

**DOI:** 10.1016/j.isci.2024.110440

**Published:** 2024-07-02

**Authors:** Matilda Gibbons, Elisa Pasquini, Amelia Kowalewska, Eva Read, Sam Gibson, Andrew Crump, Cwyn Solvi, Elisabetta Versace, Lars Chittka

**Affiliations:** 1 Department of Neuroscience, University of Pennsylvania, Philadelphia, PA 19104, USA; 2 School of Biological and Behavioral Sciences, Queen Mary University of London, London E1 4NS, UK; 3 Center for Mind/Brain Sciences, University of Trento, Rovereto 38068, Italy; 4 Academic Training Team, The Francis Crick Institute, London NW1 1AT, UK; 5 Department of Philosophy, Logic and Scientific Method, London School of Economics, London WC2A 2AE, UK; 6 Department of Pathobiology & Population Sciences, Royal Veterinary College, London NW1 0TU, UK; 7 Guangdong-Hong Kong-Macao Greater Bay Area Center for Brain Science and Brain-Inspired Intelligence, Southern Medical University, Guangzhou City, Guangdong Province 510515, China

**Keywords:** Zoology, Entomology, Neuroscience

## Abstract

It has been widely stated that insects do not show self-protective behavior toward noxiously-stimulated body parts, but this claim has never been empirically tested. Here, we tested whether an insect species displays a type of self-protective behavior: self-grooming a noxiously-stimulated site. We touched bumblebees (*Bombus terrestris*) on an antenna with a noxiously heated (65°C) probe and found that, in the first 2 min after this stimulus, bees groomed their touched antenna more than their untouched antenna, and more than bees that were touched with an unheated probe or not touched at all did. Our results present evidence that bumblebees display self-protective behavior. We discuss the potential neural mechanisms of this behavior and the implications for whether insects feel pain.

## Highlights

• We demonstrated that bees show a form of self-protective behavior
• Bees directed grooming toward their antenna that was touched with a heated probe
• This self-protective behavior occurred in the first 2 min after stimulation

## Graphical abstract

**Figure:**
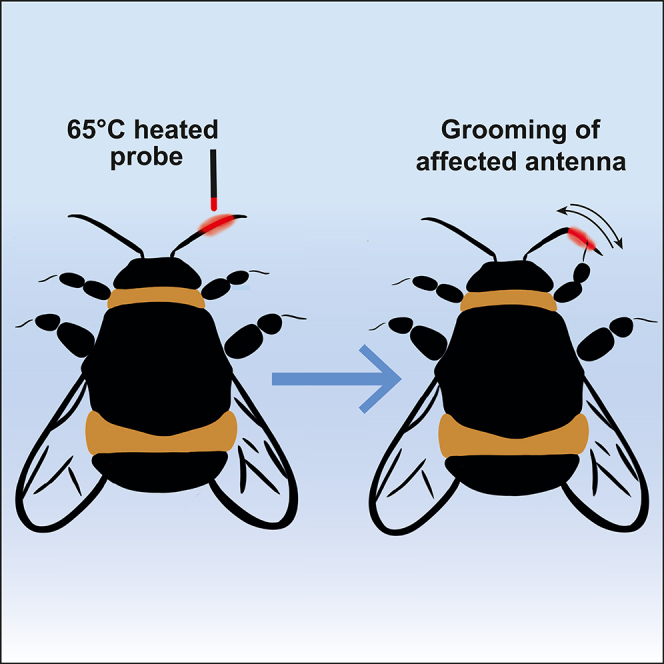

## Introduction

Nociception is the detection and processing of noxious stimuli^1^ and can be identified from recording neural activity or behavior associated with the activation of nociceptive circuits.^2^^,^^3^ Insects have both nociceptors and nociceptive neurons that detect mechanical, thermal, and chemical noxious stimuli,^4^^,^^5^ and they respond behaviorally by moving away from and avoiding noxious stimuli.^6^^,^^7^

Self-protective behavior—behavior with the aim to protect a body part from further noxious stimulation—is seen in response to noxious stimulation in many species, including humans. Examples include tending to, guarding, self-grooming, or rubbing a noxiously-stimulated body part. In humans, this can be seen, for example, when you grab and rub your bumped toe to reduce the pain caused by the nociceptive processing. In insects, there are no quantitative studies of self-protective behavior (such as self-grooming) directed toward a noxiously-stimulated site.^8^ In fact, anecdotal reports claim that insects do not protect their injury sites, and that insects continue to walk, feed, and mate normally after injury.^9^^,^^10^ These reports, alongside the lack of empirical evidence, are often cited as evidence against insects experiencing pain.^11^^,^^12^^,^^13^

In other animals, self-protective behavior is widely reported. Rats (*Rattus norvegicus*) rub their face after being injected with a noxious substance^14^ and some bird species groom limbs that have been injected with a noxious substance (e.g., *Pyrrhura molinae*^15^). There are similar findings of fish (*Oncorhynchus mykiss*) rubbing an area that was treated with a noxious injection into the gravel and the sides of their tank.^16^ Some invertebrates have also been observed performing self-protective behavior, in the form of self-grooming a noxiously-stimulated site. For example, Asian shore crabs (*Hemigrapsus sanguines*) will rub a claw that has been injected with formalin.^17^ Similarly, shore crabs (*Carcinus maenas*),^18^ prawns (*Palaemon elegans*),^19^ cuttlefish (*Sepia pharoaensis*),^20^ and octopuses (*Octopus bocki*)^21^ will groom or scratch a body part where acetic acid has been applied. The latter will also respond with self-grooming an area on their arm after it was squeezed with serrated forceps for up to 20 s.^22^

As noted previously, there is no evidence of insects self-grooming noxiously-stimulated body parts. However, insects are known to self-groom in non-noxious contexts, for example during general cleaning,^23^ and when removing dust particles (e.g., in the German cockroach *Blattella germanica*^24^), pollen grains (e.g., in bees^25^), and parasites such as mites (e.g., in honeybees, *Apis mellifera*^26^). Further, after noxious stimulation, insects may also generally groom themselves more all over, or change their grooming pattern. For example, after having their antenna amputated, red mason bees (*Osmia bicornis*) groom their head and body, although, in the study where this was found, no site-specific measurements were found/taken, nor was there a non-noxious control to compare to.^27^

Some anecdotal reports claim that insects do not protect their injury sites,^9^^,^^10^ but there are also some reports suggesting the contrary. Such reports, however, have not yet been supported by quantitative or statistical analyses.^8^^,^^28^ For example, when pinched on the abdominal proleg, moth larvae (*Manduca sexta*) reportedly turn their heads to the wound, and repeatedly touch the area with their mouthparts, but this behavior was not measured or compared to a control.^29^ The same was the case in a study on cockroaches (*Periplaneta americana)* which were reported to groom their wounds following an abdominal puncture.^30^ Since both reports of the absence and the existence of self-protective behavior in insects are not supported by quantitative measurements or analyses,^28^ a robust, experimental assessment of self-protective behavior in response to noxious stimuli in insects is required. The absence of quantitative empirical studies of whether insects perform self-protective behavior has fueled arguments against insects feeling pain, based on the claim that they do not protect their injury sites.^9^^,^^10^^,^^11^^,^^12^^,^^13^

In this study, we tested whether *Bombus terrestris* bumblebees display a type of self-protective behavior: selectively grooming a noxiously-stimulated body part. For each bumblebee, we either briefly touched one antenna with a noxious stimulus (a 65°C heat probe), or a non-noxious tactile stimulus (an unheated probe), or we did not touch either antenna (control). We recorded self-grooming behavior toward both antennae for 25 min. If bees specifically groom a site of noxious stimulation, we would predict more grooming on the noxiously-stimulated antenna than the other antenna. We would not expect this difference in bees touched with an unheated probe, nor by bees that were not touched.

## Results

### Self-grooming in the 25-min observation period

**Figure 1:**
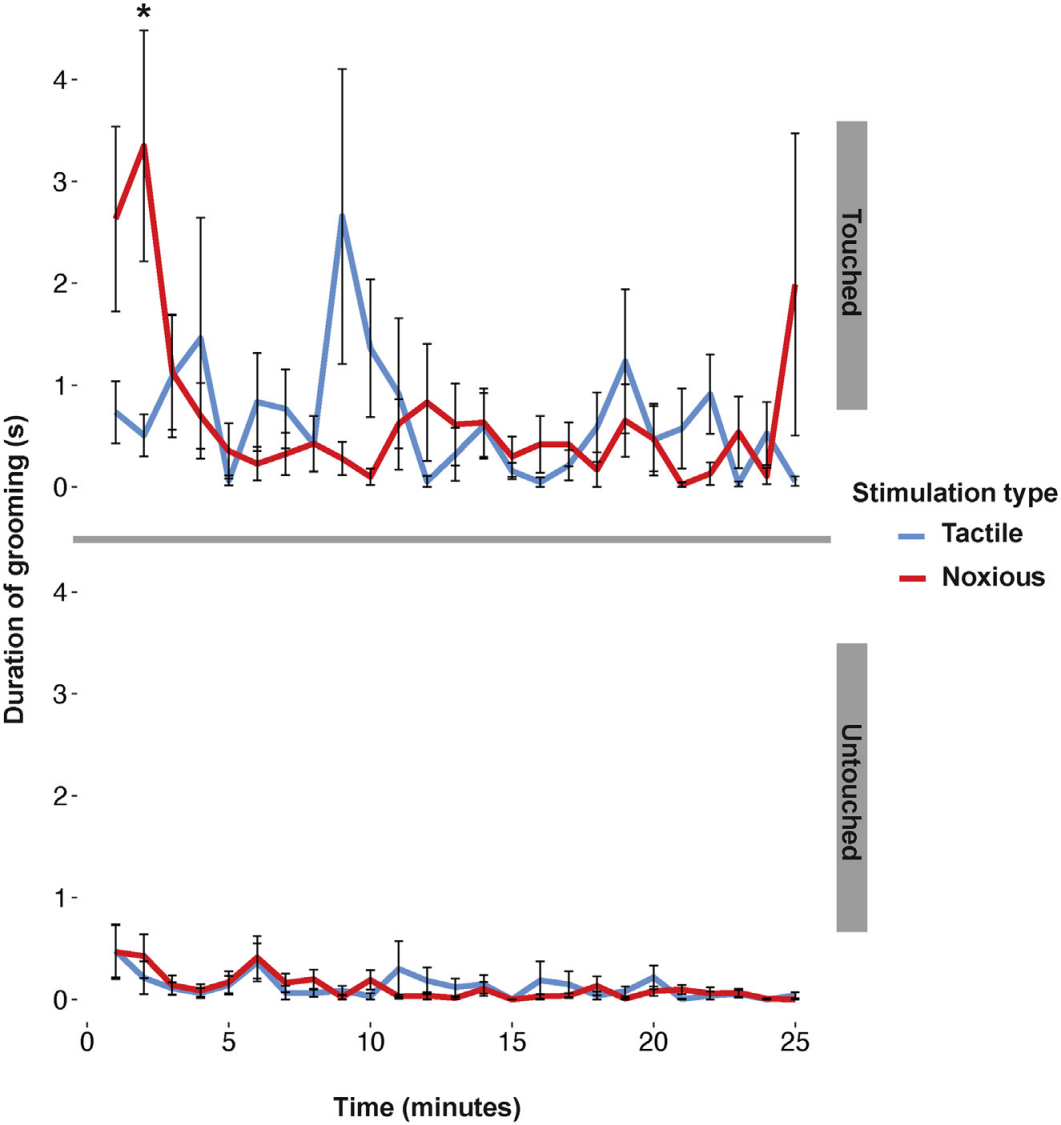
Mean duration of grooming for the untouched and touched antenna per each minute after noxious or tactile stimulation ∗*p* < 0.001; Wilcoxon test. Error bars represent the standard error of the mean.

We first tested whether, within the whole 25-min observation period, there was a difference between grooming durations on the touched and untouched antennae, and, if so, whether this difference was larger when the probe was noxiously heated. For the 25-min observation period, bees groomed their touched antennae significantly more (touched: 18.11 ± 26.79 s; untouched: 2.22 ± 3.57 s; t_5792_ = 5.922; *p* < 0.001; *N* = 40), regardless of whether the stimulation was noxious or non-noxious tactile (no significant effect: t_5792_ = 0.056, *p* = 0.955; *N* = 40; no significant interaction: t_5792_ = −0.224, *p* = 0.822; *N* = 40; Figure 1). Therefore, over the 25 min, grooming was directed toward the touched antenna, but not the noxiously-stimulated antenna specifically.

We also observed a significant interaction effect of sex on the total grooming duration over the 25 min, with workers (females) grooming their touched antenna (and not their untouched antenna) for significantly longer than males (females: *N* = 40; touched antenna: 22.89 ± 30.29 s; untouched antenna: 2.60 ± 3.78 s; males: *N* = 18; touched antenna: 7.59 ± 11.33 s; untouched antenna: 1.36 ± 2.96 s; t_5792_ = −2.665; *p* < 0.01).

### Self-grooming in the 2 mins after manipulation

**Figure 2:**
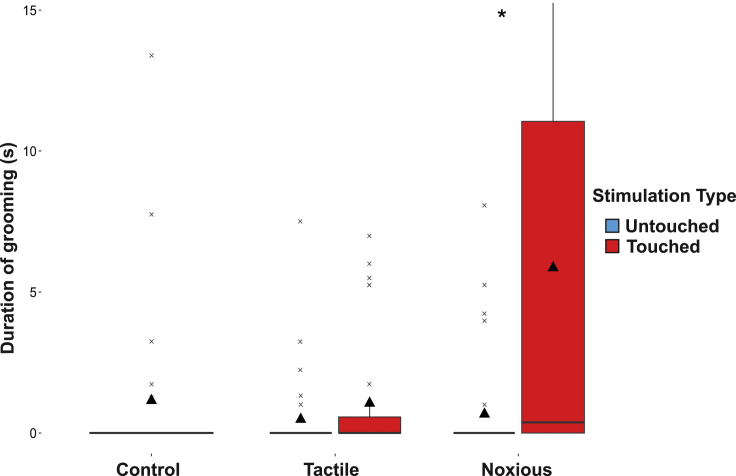
Boxplot of duration of grooming on each antenna for each stimulation type group Boxplot boundaries indicate the 25th and 75th percentiles; the whiskers indicate the minimum and maximum values within 1.5 times the interquartile range. Crosses indicate values outside this range (boxplot outliers); triangles indicate the mean; lines indicate the median. ∗*p* < 0.001; Wilcoxon test.

In the 0–2 min time bin (the only time bin with a significant *p* value after applying the Holm-Bonferroni correction, see method details), bees groomed the touched antenna more than the untouched antenna when the touch was noxious (significant interaction: t_459_ = 3.069, *p* < 0.005; *N* = 40). This result was further supported by Wilcoxon tests: in this time bin, noxiously-stimulated bees groomed their touched antenna (6.65 ± 8.8 s) significantly more than their untouched antenna (0.75 ± 1.95 s; W = 249.5, *p* < 0.001; *N* = 30; Figure 2). By contrast, for tactilely-stimulated bees, there was no significant difference in grooming between the touched antenna (1.19 ± 2.23) and the untouched antenna (0.55 ± 1.57 s; W = 324, *p* = 0.159; *N* = 28; Figure 2). Similarly, noxiously-stimulated bees groomed for significantly longer than the tactilely-stimulated bees on the touched antenna (t_83_ = 2.885, p < 0.005; N = 40; Figure 2), but not on the untouched antenna (t_83_ = 0.647, p = 0.519; N = 40; Figure 2). There was no significant effect of sex on either the grooming in touched or untouched conditions (touched: t_83_ = 0.253, p = 0.800; N = 40; untouched: t_83_ = -1.273; p = 0.207; N = 40) nor on the overall antennal grooming in noxious and tactilely-stimulated bees (t_459_ = -0.851, p = 0.395; N = 40).

We then tested whether the duration of antennal grooming was greater for either the noxiously-stimulated or the tactilely-stimulated bees compared to the control (untouched) bees. Noxiously-stimulated bees groomed their touched, and not their untouched, antenna for longer than the control bees groomed either antenna (touched: 2.85 ± 5.48 s; t_75_ = 2.55, *p* = 0.0127; *N* = 54; untouched: 0.50 ± 1.64; t_75_ = −0.318, *p* = 0.752; *N* = 54; either antenna (mean grooming duration): 0.57 ± 2.14; Figure 2). There was no significant effect of sex on either the grooming compared to control in touched or untouched conditions (touched: t_75_ = −0.111, *p* = 9.117; *N* = 54; untouched: t_75_ = −1.493; *p* = 0.140; *N* = 54). By contrast, tactilely-stimulated bees did not groom either their touched or untouched antennae for significantly longer than the control bees groomed either antenna (touched: 0.77 ± 1.84; t_73_ = −0.404, *p* = 0.689; *N* = 52; untouched: 0.48 ± 1.54; t_73_ = 0.228, *p* = 0.821; *N* = 52; Figure 2; either antenna (mean grooming duration): 0.57 ± 2.14). There was no significant effect of sex on either the grooming compared to control in touched or untouched conditions (touched: t_75_ = −0.127, *p* = 0.210; *N* = 52; untouched: t_75_ = −1.875; *p* = 0.065; *N* = 52).

## Discussion

Our results provide the first quantitative evidence of self-protective behavior in insects. In the first 2 min after noxious stimulation on an antenna, bees groomed this noxiously-touched antenna more than their untouched antenna and more than control (untouched) bees groomed either of their untouched antennae. The same results were not found in bees that were touched with a non-noxious, tactile stimulus. Further, noxiously-stimulated bees groomed their noxiously-touched antenna for longer than the tactilely-stimulated bees groomed their tactilely-touched antenna.

A significant increase in self-grooming the noxiously-stimulated antenna only being evident in the first 2 min after stimulation is consistent with studies on other invertebrates, which describe self-grooming in the first few minutes after noxious stimulation.^17^^,^^18^^,^^19^ A reason for this timing might be that the nociceptive processing ceased after around 2 min; this would likely change with a higher intensity of the noxious stimulus than we used here. An association between grooming and the cessation or onset of nociceptive processing has been previously noted in mice, in response to nociceptive formalin injection. There is an acute grooming phase, which apparently relates to the injection itself and lasts 3 min, then no grooming is seen for another 3 min, followed by a tonic phase that is longer-lasting and appears to correspond to formalin’s inflammatory effects.^31^^,^^32^^,^^33^ By analogy, we suggest that, in our study, the first 2 min corresponded to an acute phase of grooming in response to the noxious heat stimulation. Based on this evidence, future research should investigate the neural processing of noxious heat stimulation in insects, and how the temporal characteristics of the self-grooming might relate.

If grooming directed toward a noxiously-stimulated antenna happens in the first 2 min after stimulation, one might expect to also find a significant increase in grooming within the first minute. Here, we did observe an increase in grooming on the noxiously treated antenna in the first minute, but this increase was not statistically significant after correcting for multiple comparisons (Figure 1). This could reasonably be explained by our use of the Holm-Bonferroni correction, which has a high risk of false negatives.^34^

In the first 25 min after stimulation, the bees’ sex had a significant effect on how long they groomed their touched antenna, regardless of whether the stimulus was noxious or not, with workers (females) grooming their touched antenna for longer on average than males did. There are currently no studies investigating sex differences in self-grooming behavior in bees, but male bees do not groom pollen off their bodies, suggesting that they might be less equipped for self-grooming in response to something touching their body.^35^

An interesting future line of research would involve investigating the neural underpinnings of our findings. The neural processing of the noxious heat might be similar to that seen in honeybees, where nociceptive signals in the antennae are detected by thermo-sensory neurons and carried to the antennal lobe.^36^ As for the neural circuits of self-grooming, these have, of course, only been studied in the context of general, non-noxious self-grooming. For example, research on *Drosophila melanogaster* has identified neurons in the antenna that project to the ventral brain and antennal descending neurons that, if stimulated, cause antennal grooming.^37^ Nociceptive self-grooming in bees might use similar neural mechanisms, but more research is needed.

What might our results mean for the topic of insect pain? Firstly, we need to clarify whether and how self-protective behavior might relate to pain. Self-protective behavior has been taken as evidence consistent with the presence of pain in other animals (e.g., crustaceans,^17^ molluscs,^20^^,^^21^^,^^22^ rodents,^14^ birds,^15^ and fish^16^), including humans^38^ and is included in frameworks for assessing pain in animals.^39^^,^^40^^,^^41^ One reason for this association is that self-protective behavior seems to reduce the feeling of pain in humans^42^^,^^43^ and is not merely a reflexive behavior. For example, self-touch has been found to reduce the painful perception of heat, even when this “heat pain” is caused by an illusion that leads participants to perceive pain without there being any nociceptive stimulus.^44^ This shows that self-touch reduces pain specifically, rather than nociceptive processing.

There are, however, some historic studies in frogs and dogs with severed spines where noxious stimulation of extremities induces leg movements that are roughly directed toward the site of stimulation, suggesting that nociceptive reflexes might underlie some sort of self-protective behavior.^45^^,^^46^ However, the animal pain frameworks clarify that self-protective behavior should be directed toward the injury site^40^^,^^47^ and, in these studies, the leg movements are not directed specifically to the site of noxious stimulation. This might mean that general self-grooming in response to injury might be able to occur via nociceptive reflex loops in the spinal cord, but directing the response specifically to the site of injury may require some sort of brain processing. It should also be noted that these studies lack solid experimental measures, such as quantified behavior, mention of sample size, formal analysis, or a control experiment using non-noxious stimuli or healthy animals, so the results cannot be directly compared to our study. Moreover, it is clear that the behavior we observed requires the brain, since noxious stimulation of the antenna feeds directly into the antennal lobe of the bee brain.^36^

In conclusion, the self-protective behavior displayed by the bees in our study both requires the brain and is akin to a behavior that is associated with pain in humans and other animals. What does this mean for the likelihood that bees can feel pain? Our study shares with others (including those on vertebrates) the challenge that it is currently impossible to be certain about whether a behavior includes the affective component of pain. Therefore, to assess whether an animal can feel pain, it is valuable to collect evidence from multiple different lines of neural, behavioral, and psychological investigations to shift probabilities for or against.^39^^,^^40^^,^^48^ Self-protective behavior is included as one of eight criteria for the evidence of pain in other animals.^40^ Before our study, Adult Hymenoptera already fulfilled four of these eight indicators of pain, namely they have nociceptors^49^ and sensory integrative brain regions,^50^ display motivational trade-offs,^51^ and learn from aversive experiences.^52^ Our study thus provides evidence for a 5th criterion, self-protective behaviour, so Hymenoptera might now be considered in this framework to show “strong evidence for pain.”

Therefore, our study is one of a set of studies that have found evidence of traits indicative of pain in Hymenoptera. These studies, when taken together, give reason to increase our confidence that bees may feel pain. Further, at the very least, our results are incompatible with an often-quoted argument against the existence of pain in insects—the (empirically unsubstantiated) claim that they lack any form of self-protective behavior in response to noxious stimulation.^9^^,^^10^^,^^11^^,^^12^^,^^13^

### Limitations of the study

We suspect that the self-grooming we observed with this set-up may only be a fraction of the bees’ natural response, when not under stress or in a novel environment, since stress and novel contexts have been found to reduce the expression of behaviors after noxious stimulation in insects (honeybees^53^), similarly to other taxa (humans^54^; rodents,^55^ fish,^56^ birds,^57^ and snails^58^). The experiment contained multiple novel and/or potentially stressful experiences and environments for the bees. For example, the stimulation itself involved them climbing onto metal forceps, being lifted out of the nest box, and immobilized during the stimulation—all potential stressors. Further, bees were isolated from the nest and other colony members during testing, and their normal route back to the nest was blocked. In future experiments, observing bees in the nest post-stimulation may lead to the identification of their more naturalistic behavior in response to noxious stimulation.

## STAR★Methods

### Key resources table

**Table:** 

REAGENT or RESOURCE	SOURCE	IDENTIFIER
**Deposited data**		

Behavioral data	Figshare	https://doi.org/10.6084/m9.figshare.24534622.v1
Code	Figshare	https://doi.org/10.6084/m9.figshare.24498304.v3

**Experimental models: Organisms/strains**		

*Bombus terrestris* bees	Biobest Group, Belgium	NA

**Software and algorithms**		

R Studio	R Core Team, Cran-*r*-project, Vienna, Austria, version 2022.12.0 + 353	NA
BORIS	Version 7.9.15; Italy	NA

### Resource availability

#### Lead contact

Further information and requests for resources and reagents should be directed to and will be fulfilled by the lead contact, Matilda Gibbons (matilda.gibbons@pennmedicine.upenn.edu).

#### Materials availability

This study did not generate new unique materials.

#### Data and code availability

• Grooming duration data have been deposited at Figshare and are publicly available as of the date of publication. DOIs are listed in the key resources table.
• All original code has been deposited at Figshare and is publicly available as of the date of publication. DOIs are listed in the key resources table.
• Any additional information required to reanalyze the data reported in this paper is available from the lead contact upon request.

### Experimental model and study participant details

We used 82 adult bees from seven bumblebee colonies (standard hives from Biobest Group, Belgium). The bees were group-housed in ventilated wooden boxes (56 × 16 × 11 cm; see below figure). Each box comprised four sections, arranged linearly and connected by 1cm-diameter holes. At one end was the section containing the nest, which was covered with plywood. The section at the opposite end contained a 35 mL cylindrical feeder (74.5 × 31 mm), which dispensed Biogluc sugar solution *ad libitum* (Biobest group, Belgium). To access the food source, the bees had to cross the middle two sections. The middle section adjacent to the feeding section was the observation box during the testing period. The floor of both middle sections was covered with a thin layer of cat litter (Catsan Hygiene Plus, Mars Inc, USA) to absorb waste and debris. Each colony received 7g of pollen (Natupol Pollen, Koppert Biological Systems) every two days, and the laboratory was maintained at 23°C. We sexed each bee visually post-testing from the videos, based on the presence (in females) or absence (in males) of a black abdomen tip. There were 40 females and 18 males. The effect of sex on grooming behavior is discussed in the results. Bees from the same colony were pseudo-randomly assigned to experimental groups (pseudo-random to ensure there were bees in each experimental group from each colony).

**Figure:**
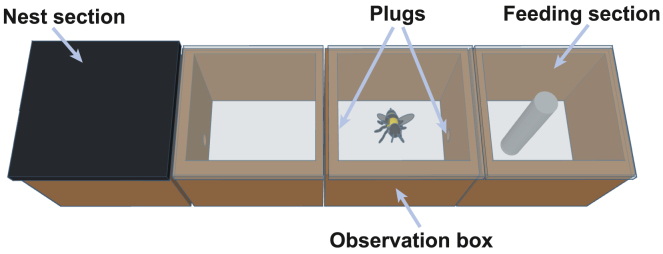
Housing and testing apparatus A ventilated wooden box (56 × 16 × 11 cm) with four sections. The nest section was covered with plywood. The feeding section contained a feeder with *ab libitum* food. The observation box was adjacent to the feeding section. Rubber plugs prevented the bee from leaving the observation box during the experiment.

### Method details

#### Treatments

The United Kingdom does not regulate insect welfare in research. Nonetheless, we followed the 3Rs principles^59^ in our experimental design and husbandry. In this vein, although some noxious stimulation is required to study self-protective behavior, we chose a temperature that, when brief, has no long-term effects on bees (65°C; based on^60^). We also used a power analysis to estimate the minimum required sample size (estimated sample size = 80; alpha: 0.05; power: 80%). According to current best practice, we have followed the ARRIVE guidelines for reporting this research.^61^

For testing, we removed bees individually from the nest box by letting them walk onto metal forceps and placed them into a marking cage (Thorne, UK). A sponge in the marking cage was used to temporarily immobilize the bees to ensure precisely targeted noxious stimulation. A soldering iron (HAKKO FX-888D; Japan) was either heated to 65°C (noxious condition) or not heated (tactile condition), then touched onto the right or left antenna (counterbalanced across bees) for 5 s. We chose this method of noxious stimulation based on how stimulation of a honeybee’s (*Apis mellifera)* antenna with a 65°C heat probe causes consistent sting extension reflexes^62^ (a defense reflex seen in response to noxious stimuli^52^). Thirty bees were touched with the noxiously heated probe (noxiously stimulated; *N* = 30); 28 were touched with the control unheated probe (tactilely stimulated; *N* = 28); and 24 were put in the marking cage but not touched with a probe (control: *N* = 24). No bees were excluded from the analysis. We used an RST Soldering Iron Tip Thermometer 191 (YWBL- WH; China) to ensure the correct temperature of the soldering iron. After the treatment, bees were immediately placed in the observation box and filmed with an iPhone 8 (Apple; USA) for 25 min. We sealed the holes between boxes during the experiment, so bees were confined to the observation box (14 × 16 × 11 cm).

#### Behavioral analysis

Four treatment-blind coders recorded the self-grooming behavior displayed in the 25-min videos using BORIS behavioral analysis software (BORIS, version 7.9.15; Italy). Self-grooming was defined as ‘the right or left front, middle, or hind leg moves over the left or right antenna either in one direction or in a repeated back and forth motion’. To measure inter-rater reliability, all four raters recorded grooming behavior for two bees (corresponding to two 25-min videos: one noxiously stimulated bee and one tactilely stimulated bee). Because the rating scale was continuous, we calculated the intraclass correlation coefficient. The correlation compared the total grooming duration of the right and left antenna across the four raters. The coefficient was 0.86, on a scale of 0–1, indicating a ‘good’ reliability.^63^

### Quantification and statistical analysis

We analyzed the data in R Studio (R Core Team, Cran-*r*-project, Vienna, Austria, version 2022.12.0 + 353), using general linear mixed effect models (GLMMs; packages: ‘lme4’ and ‘car’) and Wilcoxon tests. We checked model assumptions using histograms and ‘Q-Q plots’, and corrected for multiple testing using the Holm-Bonferroni correction.^64^ We considered *p* < 0.05 significant and define N as number of bees. Sample size was determined by a power analysis (estimated sample size = 80; alpha: 0.05; power: 80%). Statistical details of the experiments can be found in the results section and in Figure 2.

To test for a difference between the grooming duration on the touched versus untouched antenna in noxiously stimulated and tactilely stimulated bees, we ran a GLMM. The response variable was the duration of antennal grooming for each antenna per bee. The fixed effects were stimulation type (noxious or tactile), whether the antenna was touched or untouched, the sex of the bee and their interaction. The random effect was the bee identity. We ran this model for the whole observation period (25 min), as well as individual time bins 0–1, 0–2, 0–3, 0–4 0-5, 6–10, 11–15, 16–20 and 21–25 min. We tested the individual time bins because some previous invertebrate studies have only detected self-grooming within the first few minutes after stimulation.^17^^,^^18^^,^^19^ The only time bin with a significant interaction effect (after applying the Holm-Bonferroni correction for multiple testing) was 0–2 min, so this is the only time bin we ran the other GLMM and Wilcoxon tests on (described below).

We used unpaired two-sample Wilcoxon tests (as our data did not meet the criteria for parametric analysis) to test the difference between the grooming durations on the touched or untouched antenna in the tactile and noxious treatment groups in the first 2 min after stimulation.

We ran another GLMM to test for a difference between grooming durations on either the touched or untouched antenna in the noxiously stimulated and tactilely stimulated bees, and the mean grooming duration for both antennae in bees in the control condition in the first 2 min. The response variable was either the duration of grooming on the touched antenna per bee or the duration of grooming on the untouched antenna per bee, or, for control bees, the mean grooming on one antenna was used, because neither antenna was touched in this condition. The fixed effects were the stimulation type (noxious, tactile, control) and the sex of the bee. The random effect was bee identity.
